# Emerging 2D MXene Materials for Flexible Thermoelectric Energy Harvesting

**DOI:** 10.3390/nano16040244

**Published:** 2026-02-13

**Authors:** Jiahui Li, Xiaoyu Shi, Qiudi Lu, Yang Zhang, Zhangping Jin, Binghan Dai, Bo Wu

**Affiliations:** 1 Nanjing University of Posts & Telecommunications, 9 Wenyuan Road, Nanjing 210023, China; sxy15135083669@163.com (X.S.); 18386149216@163.com (Q.L.); 18762267734@163.com (Y.Z.); 1223066444@njupt.edu.cn (Z.J.); 2 College of Foreign Studies, China University of Geosciences, Beijing 100083, China; 3 College of Materials Science and Engineering, City University of Hong Kong, Hong Kong, China

**Keywords:** MXenes, TE materials, energy harvesting, flexible electronics, surface engineering

## Abstract

The pursuit of energy-efficient technologies is crucial for achieving sustainability amid rising global energy demands and climate concerns. MXenes—a class of two-dimensional (2D) transition metal carbides, nitrides, and carbonitrides—have recently attracted significant attention in thermoelectric (TE) research due to their outstanding electrical conductivity, tunable surface chemistry, and unique layered structures. This review uniquely focuses on the integration of MXenes into flexible and wearable platforms, offering a systematic analysis of material innovations specifically tailored to mechanical compliance. Beyond material-level transport properties, we critically evaluate actual device-level demonstrations, including fabrication strategies for flexible TE generators (f-TEGs), that achieve impressive outputs, such as Seebeck voltages of up to 399.9 mV for 200 p-n modules. To assist readers in gauging progress, we provide a comprehensive comparative analysis of diverse MXene architectures, summarized in a quantitative benchmark table covering Seebeck coefficients (S), electrical conductivity (σ), power factor (PF), and ZT values. Notably, experimental optimization has led to performance breakthroughs, with MXene-based flexible films exhibiting power factors exceeding 2100 µW m^−1^ K^−2^ and ZT values as high as 1.33 at room temperature. Finally, critical challenges, including environmental stability and large-scale manufacturing, are discussed alongside future perspectives on multifunctional MXene systems.

## 1. Introduction

**Figure 1:**
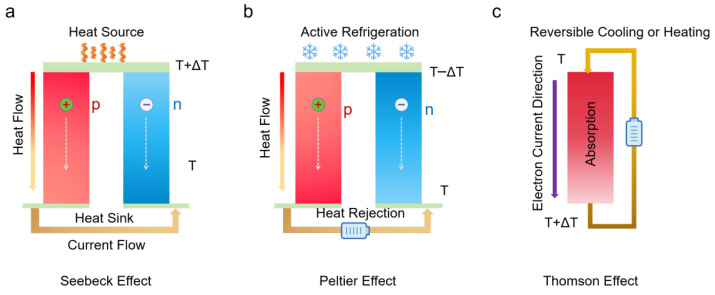
Overview of TE mechanisms, (**a**) Seebeck effect, (**b**) Peltier effect, and (**c**) Thomson effect.

As global energy consumption continues to surge, accompanied by the depletion of fossil fuels and increasing carbon emissions [1,2], the development of sustainable, decentralized, and renewable energy technologies has become a pressing priority [3,4]. Energy harvesting systems capable of capturing ambient energy—such as solar, mechanical, or thermal energy—offer a promising pathway toward energy autonomy in next-generation electronic devices [5]. Among these, TE technology, which enables the direct conversion of heat into electricity via the Seebeck effect, has gained significant attention due to its solid-state nature, scalability, and maintenance-free operation. TE generators (TEGs) are particularly attractive for low-power and miniaturized electronics, where conventional batteries face limitations in terms of weight, flexibility, and lifespan. This demand is further amplified by the rapid expansion of wearable electronics, implantable sensors, flexible displays, and Internet of Things (IoT) devices [6]. TE phenomena encompass three fundamental effects: the Seebeck, Peltier, and Thomson effects. The Seebeck effect describes the generation of an electric voltage when a temperature gradient is applied across a material, driven by the directional diffusion of charge carriers, either electrons or holes (Figure 1a). The resulting thermovoltage (ΔV) is linearly proportional to the temperature difference (ΔT), expressed as ΔV = SΔT, where S denotes the Seebeck coefficient. In contrast, the Peltier effect (Figure 1b) involves the absorption or emission of heat at the junction between two different conductors when an electric current flows through the system. The associated heat flow (Q) is determined by the current I, following the relation Q = PI, where P is the Peltier coefficient (P = TS). The Thomson effect (Figure 1c) refers to the continuous heating or cooling observed in a single, homogeneous conductor when both an electric current and a thermal gradient are present simultaneously. Collectively, these effects define the principles behind TE energy conversion. Regardless of the mechanism, the overall conversion efficiency of TE devices is governed by the material’s dimensionless figure of merit, ZT [6,7].

$$ZT=\frac{S^{2}\sigma }{\kappa }T$$

where S is the Seebeck coefficient, σ is the electrical conductivity, κ is the thermal conductivity, and T is the absolute temperature. Achieving a high ZT requires simultaneously maximizing *S* and *σ* while minimizing *κ*, a challenging task due to the intrinsic trade-offs among these parameters.

For flexible TE devices, additional material requirements include mechanical flexibility, stretchability, and integration compatibility with soft substrates and textile platforms [8]. The term *S*^2^*σ*, commonly referred to as the power factor, serves as a critical indicator of the electrical contribution to TE performance. A major challenge in developing high-efficiency TE materials lies in maximizing the PF while managing thermal transport. While TE technologies have been extensively studied, conventional high-performance materials, such as Bi_2_Te_3_, PbTe, and SnSe, are inherently rigid, brittle, and often composed of toxic elements, hindering their application in flexible or biocompatible systems. Conversely, organic semiconductors and conductive polymers offer superior mechanical compliance and solution processability, but generally suffer from low carrier mobility and limited TE efficiency. These limitations underscore the need for new material systems that synergistically combine high TE performance with mechanical adaptability [9,10].

Two-dimensional materials have become leading contenders for flexible TE devices due to their superb structural flexibility and tunable physical properties. Among them, MXenes, a family of 2D transition metal carbides, nitrides, and carbonitrides with a general formula of M_n+1_X_n_T_x_, have demonstrated remarkable potential. Here, M is a transition metal, X is carbon and/or nitrogen, and T_x_ represents surface terminations such as –O, –OH, –F, and –Cl. Their unique properties, including high conductivity, tunable band structure, and large surface area, make them ideal for TE conversion [11,12,13].

For instance, Ti_3_C_2_T_x_ films have shown metallic conductivities as high as ~2.4 × 10^3^ S cm^−1^ [14], while theoretical predictions suggest that Ti_2_CO_2_ and Sc_2_C(OH)_2_ may exhibit large Seebeck coefficients of approximately 114 μV K^−1^ and 220 μV K^−1^, respectively. The broad compositional diversity and abundant surface terminations (–F, =O, –Cl, –Br, etc.) allow for precise control over the band structure and phonon scattering mechanisms. Recent studies have highlighted that surface terminations not only modulate electronic properties, but also significantly influence thermal transport behavior, making them a key design parameter for enhancing TE performance [15,16].

While MXenes have been extensively studied for applications such as energy storage, electromagnetic interference shielding, and chemical sensing, their integration into TE systems, especially flexible TEGs, remains relatively underexplored. Although prior reviews, such as that by Xu et al. (2024) [17], have touched upon the TE potential of MXenes, there is a lack of focused, systematic analyses addressing recent material innovations and integration challenges specific to wearable TE platforms.

**Figure 2:**
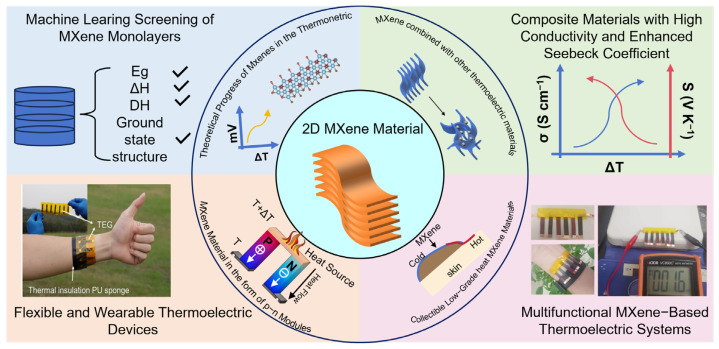
Overview of the latest research progress in 2D MXenes.

This review aims to fill that gap by providing a comprehensive summary of both the theoretical insights and experimental advances in MXene-based TE materials, with an emphasis on flexible device applications (Figure 2). We examine the key synthesis methods, structural characteristics, and the role of surface engineering in tailoring TE performance. Additionally, we explore the integration of MXenes with polymers, inorganic semiconductors, and carbon-based materials to form hybrid composites. Finally, we identify the critical challenges, including cost, scalability, and reproducibility, alongside future research directions, toward the development of high-performance MXene-based TE devices.

## 2. Theoretical TE Properties of MXenes

### 2.1. Theoretical Progress of MXenes in Thermoelectrics

**Figure 3:**
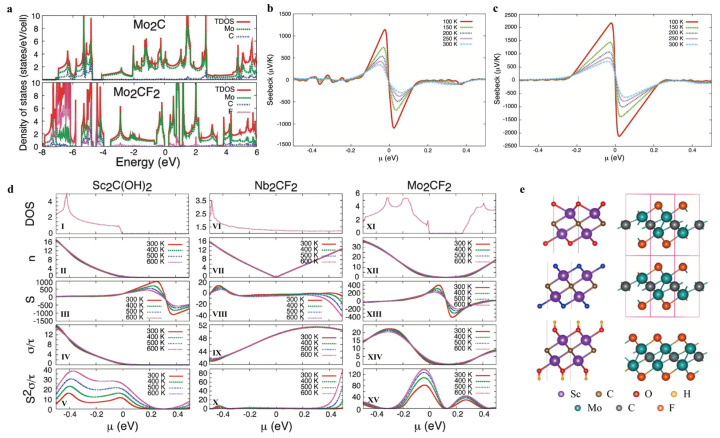
(**a**) densities of states of Mo_2_C and Mo_2_CF_2_ Copyright 2016, American Chemical Society [19]. (**b**,**c**) Predicted Seebeck coefficients for monolayer Ti_2_CO_2_ and Sc_2_C(OH)_2_. Copyright 2012, WILEY-VCH Verlag GmbH [18]. (**d**) The density of states, carrier density, the Seebeck coefficient, electrical conductivity, and the power factor illustrating the dependence on chemical potential under diverse temperature settings for the non-exfoliated forms of Sc_2_C(OH)_2_, Nb_2_CF_2_, and Mo_2_CF_2_. Copyright 2014, Royal Society of Chemistry [19]. (**e**) Cross-sectional and top-down projections of the geometric setups for diverse MXene systems, specifically functionalized Sc_2_C and pristine 2D-Mo_2_C. Copyright 2014, Royal Society of Chemistry [18,19].

The exploration of MXenes for TE applications began with foundational theoretical studies that established a baseline understanding of their intrinsic electronic and thermal transport properties. These early works laid the groundwork for subsequent innovations in material design and optimization strategies. Khazaei et al. (2012) [18] (2014) [19] were the first to investigate the TE properties of MXenes of functionalized Mo_2_C monolayers and multilayers M_2_C (M = Sc, Ti, V, Zr, Nb, Mo, Hf, and Ta) and M_2_N (M = Ti, Zr, and Hf) MXenes modified with F, OH, and O groups by implementing first-principles calculations integrated with Boltzmann transport theory (Figure 3a–d). Their results revealed that Mo_2_CF_2_ exhibits superior power factors compared to other types. Through the systematic prediction of various functionalized MXene monolayers (Figure 3e), the study provided a comprehensive classification of TE performance across different metal centers. Specifically, MXenes based on vanadium (V), niobium (Nb), and tantalum (Ta) were found to have good electrical conductivity; however, their TE efficiency is relatively low due to their negligible S about 20 μV K^−1^. Titanium (Ti)-, zirconium (Zr)-, and hafnium (Hf)-based MXenes were classified as moderate performers, while those composed of molybdenum (Mo) or nonmagnetic semiconducting chromium (Cr) demonstrated the most favorable TE properties. Notably, Mo_2_CF_2_, with a narrow bandgap of approximately 0.25 eV, was identified as the most promising compound among the materials studied.

**Figure 4:**
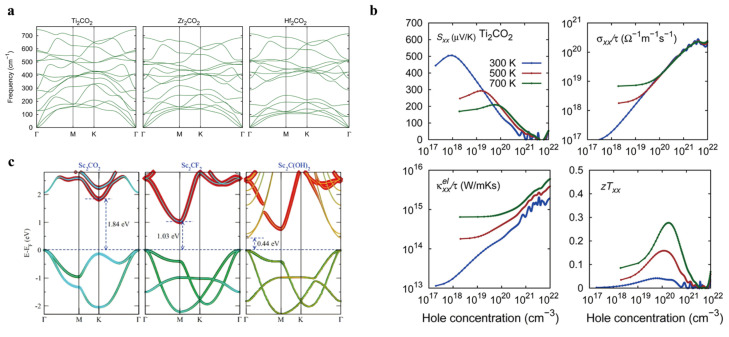
(**a**) Electronic band structures and densities of states of the MXenes M_2_CO_2_ (M = Ti, Zr, or Hf). Copyright 2016, American Chemical Society [18]. (**b**) Carrier-density-dependent variations in the electronic transport metrics (take the Ti_2_CO_2_ system as an example). Copyright 2016, American Chemical Society [20]. (**c**) electronic band structures of Sc_2_CT_x_ (Tx = O, F, or OH). Copyright 2016, American Physical Society [21].

Expanding on this understanding, Gandi et al. (2016) [20] investigated the TE properties of M_2_CO_2_ (M = Ti, Zr, Hf) systems and highlighted the importance of oxygen surface termination in tuning bandgaps, which were modulated within the range of 0.1–0.5 eV. Among these, Ti_2_CO_2_ exhibited the best TE performance, achieving a maximum ZT value of 0.27 at 700 K. Phonon lifetime analysis (Figure 4a) revealed that Ti_2_CO_2_ possessed the lowest lattice thermal conductivity, while Hf_2_CO_2_ had the highest across the 300–700 K temperature range. However, the performance of these materials is sensitive to carrier concentration; when the electron concentration drops below 1.4 × 10^21^ cm^−3^, ZT values decrease significantly due to the emergence of minority carrier effects (Figure 4b). Complementing these studies, Kumar and Schwingenschlögl (2016) [21] focused on Sc_2_C-based MXenes, electronic band structures of Sc_2_CT_x_ (Tx = O, F, or OH) (Figure 4c) and showed that hydroxyl (-OH) functionalization can enhance the Seebeck coefficient by approximately 30% while also reducing lattice thermal conductivity to ~15 W m^−1^ K^−1^ through increased phonon scattering.

In contrast to conventional wisdom where electron–phonon (e–p) scattering is typically negligible compared to phonon–phonon (p–p) interactions, first-principles calculations by Huang et al. (2019) [22] revealed that 2D metallic Nb_2_C MXene exhibits unusually strong e–p scattering even at low e–p coupling strength. At room temperature, the e–p scattering rate approaches that of p–p scattering, attributed to the near-resonance between the electronic excitation energy and characteristic phonon energies. This phenomenon results in a significant suppression of lattice thermal conductivity, providing a novel strategy for thermal transport engineering in metallic MXenes. Such materials, with stable and intrinsically low thermal conductivity, hold significant promise for low-temperature TE applications, particularly in flexible and miniaturized device architectures.

These early theoretical studies collectively highlighted the critical role of surface functionalization in modulating both electronic and thermal transport properties in MXenes. As a natural progression, researchers have since delved deeper into the effects of functional groups and compositional engineering to further optimize MXene-based TE materials.

### 2.2. Effect of Surface Functionalization on TE Properties

**Figure 5:**
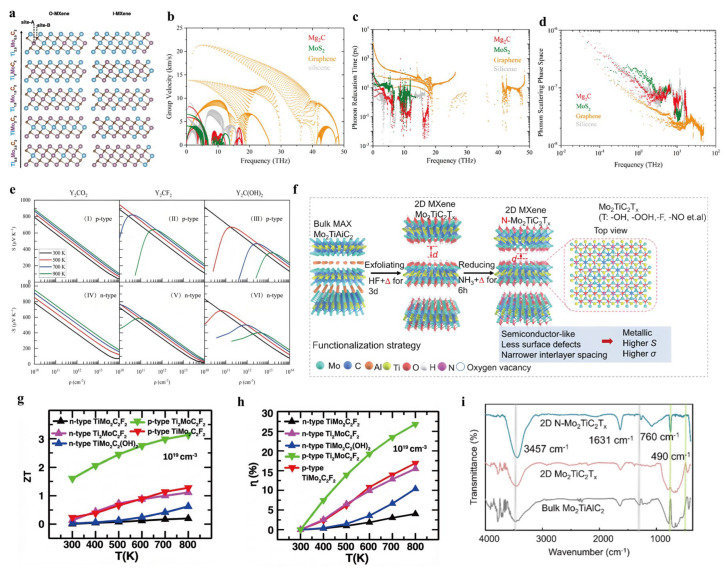
(**a**) The Ti_3−x_Mo_x_C_2_ MXenes in the O and I phase. Copyright 2020, American Physical Society [28]. (**b**–**d**) Comparative analysis of phonon lifetimes, mode-resolved group velocities, and cumulative scattering phase space for Mg_2_C (red), MoS_2_ (green), graphene (yellow), and silicene (gray) at 300 K. Copyright 2021, American Chemical Society [25]. (**e**) Carrier concentration relationship with Seebeck coefficients and TE performance metrics for p-type and n-type Y_2_CT_2_ (T ¼ O, F and OH). Copyright 2022, Royal Society of Chemistry [26]. (**f**) Functionalization strategy of heat-ammoniated Mo_2_TiC_2_T_x_ into N–Mo_2_TiC_2_T_x_. Copyright 2025, American Chemical Society [29]. (**g**,**h**) TE energy conversion performance in the identical temperature span. Copyright 2020, American Physical Society [28]. (**i**) FTIR spectra of bulk Mo_2_TiC_2_T_x_, Mo_2_TiC_2_T_x_, and ammoniated N–Mo_2_TiC_2_T_x_. Magnification is indicated by 2 μm and 500 nm scale bars for the respective images in (**c**,**d**). Copyright 2025, American Chemical Society [29].

Building early insights into MXene surface chemistry, researchers have extensively employed functionalization strategies to optimize TE performance, particularly by reducing lattice thermal conductivity and enhancing electronic transport. Among the earliest examples, Guo et al. (2018) [23] used density functional theory (DFT) to demonstrate that oxygen functionalization on Ti_2_C MXenes increases the Seebeck coefficient to approximately 350 μV K^−1^ and reduces the thermal conductivity by up to 50% (to ~10 W m^−1^ K^−1^). This improvement is attributed to the shorter phonon relaxation time and the emergence of a metal-to-semiconductor transition, indicating significant potential for TE applications. By comparison, surface termination with fluorine or hydroxyl groups leads to an increase in both lattice and electronic thermal conductivities, which may hinder TE performance. Further studies have supported the effectiveness of oxygen- and hydroxyl-based terminations. Sarikurt et al. (2018) [24] analyzed Ti_2_CO_2_ and Ti_2_CF_2_, showing that -OH and -O terminations push lattice thermal conductivity under 8 W m^−1^ K^−1^ at 300 K due to strong anharmonic interactions. Wang et al. (2021) [25] reported that monolayer Mg_2_C exhibits an ultralow thermal conductivity of approximately 2 W m^−1^ K^−1^ at 300 K, also driven by significant anharmonic phonon scattering (Figure 5b–d). In a subsequent study, Wang et al. (2022) [26] found that Y_2_CT_2_ (T = O, F, OH) can achieve Seebeck coefficients above 300 μV K^−1^ and lattice thermal conductivities below 5 W m^−1^ K^−1^ (Figure 5e), resulting from the intensified phonon scattering triggered by surface functional groups. These findings clearly demonstrate that surface functionalization plays a pivotal role in suppressing the lattice’s thermal conductivity and enhancing electronic transport. Encouraged by these functionalization-induced improvements, researchers have also begun exploring more complex MXene architectures to further optimize TE properties.

For instance, Jing et al. (2019) [27] investigated double-transition metal MXenes such as Cr_2_TiC_2_T_2_(T = –OH, –F), reporting impressive TE figures of merit (ZT) for hole-doped Cr_2_TiC_2_(OH)_2_, with values of 2.58 at room temperature and 3.00 at 600 K. These high ZT values correspond to an energy conversion efficiency of up to 20%. Similarly, Karmakar and Saha-Dasgupta (2020) [28] evaluated ordered dual-transition metal MXenes represented by the formula Ti_3−x_Mo_x_C_2_T_2_ (x = 0.5–2.5). Among the various compositions studied, Ti_2_MoC_2_F_2_, TiMo_2_C_2_F_2_, and TiMo_2_C_2_(OH)_2_ exhibit semiconducting behavior, suggesting strong potential as high-performance TE materials. Particularly, p-type doped Ti_2_MoC_2_F_2_ shows a ZT value exceeding 1 across a broad temperature range of 300–800 K, reaching a peak of 3.1 at 800 K, which corresponds to an energy conversion efficiency of approximately 27% (Figure 5g,h). Li et al. (2025) [29] propose a strategy of heat treatment to synergistically regulate the surface oxygen defects and microstructure of Mo_2_TiC_2_T_x_ o-MXene, resulting in a high conductivity and high thermoelectric power factor (Figure 5f). To investigate whether ammonia treatment modifies the MXene’s surface, we tested the Fourier transform infrared (FTIR) spectra of three different samples: bulk Mo_2_TiAlC_2_, 2D Mo_2_TiC_2_T_x_, and 2D N–Mo_2_TiC_2_T_x_ (Figure 5i).

### 2.3. Novel MXene Structures for TE Optimization

The success of surface functionalization in reducing lattice thermal conductivity has inspired researchers to explore novel MXene architectures—such as Janus MXenes, nitrogen-containing MXenes, and dual transition metal systems—as promising strategies to further enhance TE performance through compositional and structural engineering. For instance, Rana et al. (2023) [30] investigated Janus MoWCO_2_ monolayers and reported promising TE properties. At 700 K, the power factor reached 6.5 × 10^3^ μW m^−1^ K^−2^ for p-type and 1.5 × 10^3^ μW m^−1^ K^−2^ for n-type carriers. The corresponding ZT value for p-type MoWCO_2_ was approximately 0.04 at a carrier concentration of ~4 × 10^13^ cm^−2^, while the n-type ZT was nearly four times lower due to relatively high lattice thermal conductivity. Expanding beyond Janus structures, Yan et al. (2023) [31] examined nitrogen-containing MXene monolayers of X_3_N_2_O_2_ (X = Hf, Zr), explicitly incorporating electron–phonon coupling in their simulations. Due to their comparable geometric configurations, electronic band profiles, and phonon dispersion curves, these compounds exhibited balanced charge and heat transport. Notably, the conduction bands showed multi-valley characteristics, favoring n-type conduction. Maximum n-type power factors of 3.2 × 10^4^ µW m^−1^ K^−2^ and 2.3 × 10^4^ µW m^−1^ K^−2^ were achieved for Hf_3_N_2_O_2_ and Zr_3_N_2_O_2_, respectively. Hf_3_N_2_O_2_ also demonstrated a higher peak of ZT of 0.36 at 700 K, outperforming Zr_3_N_2_O_2_ (ZT = 0.15), primarily due to its lower κₗ.

**Figure 6:**
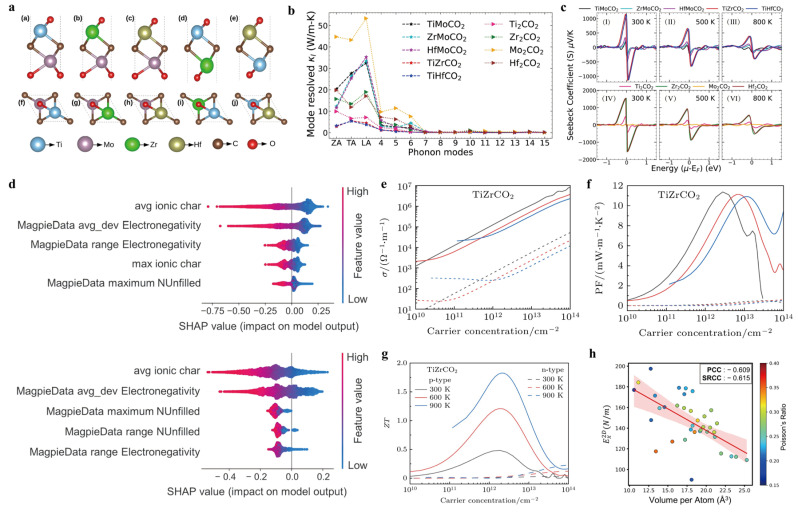
(**a**) Structural stability and lowest-energy models of Janus MXenes. (TiMoCO_2_, ZrMoCO_2_, HfMoCO_2_, TiZrCO_2_ and TiHfCO_2_). Copyright 2024, Royal Society of Chemistry [32]. (**b**) Mode-level lattice thermal conductivity for the various MXene systems. The 3 acoustic and 12 optical modes in sorted by frequency and are mapped on the horizontal axis. Copyright 2024, Royal Society of Chemistry [32]. (**c**) Seebeck coefficient plotted against energy at various temperature regimes. The top (bottom) panel represents Janus (Parent) MXenes. Copyright 2024, Royal Society of Chemistry [32]. (**d**) Relationship between volumetric occupancy and mechanical attributes: correlation between MXene thickness and mechanical attributes. Copyright 2024, Royal Society of Chemistry [34]. (**e**–**g**) Variations in monolayer TiZrCO_2_ conductivity and power factor with carrier concentration for p and n types. Copyright 2024, Chinese Physical Society & Institute of Physics, Chinese Academy of Sciences [33]. (**h**) Five key features for estimating DH of 2D materials in C2DB and MXenes in a NANt database that were extracted through Shapley Additive Explanations. Copyright 2024, Royal Society of Chemistry [34].

The impact of symmetry lowering on the interplay between electronic and thermal transport—and thus on TE performance—was further explored by Himanshu et al. (2024) [32]. In parent M_2_CO_2_ MXenes, symmetry was reduced by substituting the transition metal atom on a single surface face, leading to Janus compounds denoted as MM’CO_2_ (Figure 6a). Their calculations revealed that such surface engineering can significantly enhance the TE figure of merit. Detailed analysis attributed this improvement to changes in the electronic band structure and enhanced phonon anharmonicity caused by bond strength disparities arising from the broken symmetry. Among the compounds examined, TiZrCO_2_ and TiHfCO_2_ were predicted to achieve notably high ZT values approaching 3, surpassing those of their symmetric M_2_CO_2_ counterparts. Building on these findings, the researchers further applied biaxial tensile strain to three Janus structures—Zr_2_COS, ZrHfO_2_, and ZrHfCOS—to explore the synergistic effects of structural asymmetry and mechanical modulation. This approach led to a significant reduction in lattice thermal conductivity due to intensified phonon scattering, as well as a considerable enhancement in the Seebeck coefficient. Among the tested materials, n-type ZrHfCO_2_ exhibited the most outstanding TE performance, with a peak ZT of 3.2 at 800 K. The other two Janus compounds also showed marked improvements, achieving strain-enhanced ZT values approaching 2 (Figure 6b,c). To further boost TE performance, researchers have investigated dual-transition metal MXenes. Huang et al. (2024) [33] systematically studied the TE behavior and stability of two such monolayers: TiZrCO_2_ and VYCO_2_. Both exhibited excellent transport characteristics. At 300 K and the optimal carrier concentration, p-type TiZrCO_2_ achieved a significantly higher power factor of 1.14 × 10^4^ µW m^−1^ K^−2^ compared to its n-type counterpart. Moreover, both TiZrCO_2_ and VYCO_2_ displayed intrinsically low lattice thermal conductivities—5.08 and 3.22 W m^−1^ K^−1^ at 300 K, respectively—which further decreased to 2.14 and 1.09 W m^−1^ K^−1^ at 900 K, highlighting their potential for high-temperature TE applications (Figure 6e–g). Overall, these findings highlight the substantial possibilities of structural and compositional engineering in unlocking superior TE performance in emerging MXene systems.

Beyond structural innovations, efforts have also been made to effectively harness MXenes for TE applications using data-driven approaches. Park et al. (2024) [34] An integrated machine learning (ML) screening protocol was developed to search for stable MXene candidates while simultaneously assessing their mechanical properties. Among 23,857 possible MXene compositions, only 48 candidates were predicted to be stable. Notably, MXenes featuring halogen surface ends and fourth-group transition metals were both mechanically resilient and thermodynamically favorable. These findings suggest that the electronegativity disparity between internal elements plays a fundamental role in steering the stability and physical properties of these 2D materials (Figure 6d,h).

Wu et al. (2024) [35] pushed the boundaries with a Ti_2_CO_2_–bismuthene–Ti_2_CO_2_ heterostructure, reporting a ZT of ~1.2 at 600 K, with a power factor of ~2 μW m^−1^ K^−2^ and thermal conductivity of ~1.5 W m^−1^ K^−1^, showcasing the power of layered architectures. Nidhi Modi et al. (2025) [36] explored the strain engineering of ScYCCl_2_ monolayer MXene and its potential for metal-ion battery applications via DFT. The results showed a transition from an indirect to direct bandgap under 10% tensile strain and to a metallic state under 10% compressive strain. Stability under strain was validated using phonon dispersion analysis alongside molecular dynamics modeling. The work function increases with compressive strain and decreases with tensile strain. Optical properties, such as absorption coefficient and refractive index, are tunable through strain. When subjected to compressive strain, the metallic ScYCCl_2_ monolayer functions effectively as an anode for metal-ion batteries (Na, K, Li, Mg), boasting an impressive theoretical storage capacity alongside a minimal open-circuit voltage. Strain engineering effectively modulates the electronic and optical properties, offering potential for optoelectronic and energy storage applications.

## 3. Experimental Studies for MXene in Thermoelectric

Recent research on MXenes for TE applications can be broadly categorized into three areas. First, studies on pure MXenes have explored their intrinsic TE properties, revealing relatively high Seebeck coefficients in some cases; however, their typically metallic nature limits their overall efficiency. Second, MXene composites—formed by combining MXenes with materials such as SnTe or PEDOT: PSS—demonstrate significantly enhanced performance due to minimized lattice thermal conductivity alongside boosted electrical conductivity. Third, advancements in surface functionalization and flexible device design, such as TE fibers, highlight the practical potential of MXenes, although high-temperature and long-term stability remain challenges.

### 3.1. Pure MXenes and Their TE Properties

**Figure 7:**
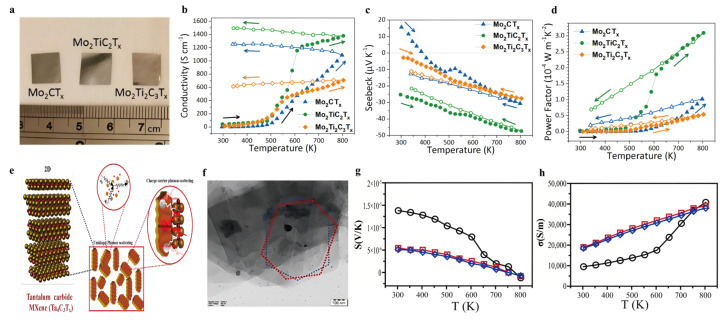
(**a**) Optical images of Mo-based MXene films. Copyright 2017, American Chemical Society [37]. (**b**) Temperature-responsive TE metrics of Mo-based MXene films during the initial thermal cycle: Electrical conductivity, (**c**) Seebeck coefficient and (**d**) TE power factor. Copyright 2017, American Chemical Society [37]. (**e**) The Tantalum carbide MXene was synthesized by two-step synthesis and schematic representation of Umklapp scattering and microstructural defect scattering, such as lattice disorder and dislocation scattering. Copyright 2024, WILEY-VCH Verlag GmbH [38]. (**f**) The Hexagonally shaped few layers shaped MXene sheets with a 90° stacking sequence as seen in the SAED pattern. Copyright 2024, WILEY-VCH Verlag GmbH [38]. (**g**) Temperature dependent Seebeck coefficient [38]. (**h**) Temperature dependent electrical properties. Copyright 2024, WILEY-VCH Verlag GmbH [38].

The journey to harness MXenes for TE applications began with the synthesis and characterization of their intrinsic properties, aiming to validate theoretical predictions and establish a foundation for material optimization. Initial efforts focused on selectively etching MAX phases to obtain 2D MXene nanosheets and tailoring their surface terminations to modulate electrical and thermal transport behavior. A landmark study by Kim et al. (2017) [37] presented the first laboratory-based evaluation of temperature-dependent TE properties for Mo-based MXenes, including Mo_2_CT_x_, Mo_2_TiC_2_T_x_, and Mo_2_Ti_2_C_3_T_x_, up to 800 K. These freestanding, binder-free films (Figure 7a)—fabricated via vacuum-assisted filtration—exhibited promising n-type TE behavior. Notably, Mo_2_TiC_2_T_x_ showed the best performance, achieving a power factor of 3.09 × 10^2^ µW m^−1^ K^−2^ at 803 K (Figure 7d), which was attributed to its high electrical conductivity (1380 S cm^−1^) (Figure 7b) and relatively large Seebeck coefficient (−47.3 μV K^−1^) (Figure 7c). The significant increase in conductivity above 500 K was ascribed to the deintercalation of water and organic molecules, along with the partial removal of surface terminations, which improved interlayer coupling. Raman analysis further confirmed the thermal robustness of these MXenes up to 800 K under an Ar/H_2_ atmosphere. This foundational work not only verified the TE viability of Mo-based MXenes, but also paved the way for further performance optimization through compositional tuning, surface engineering, and composite strategies.

Building on this, Liu et al. (2020) [13] demonstrated that implementing surface engineering is a practical way to enhance the TE metrics of films exhibiting high metallic conductivity. In a representative study, Ti_3_C_2_T_x_ MXene underwent hydrothermal treatment under varying alkali solution concentrations, temperatures, and types to modulate its surface terminations. This process led to the partial substitution of –F and –OH groups with –O terminations, adjusting the Fermi level toward the band margins alongside a simultaneous broadening of the bandgap. Cation intercalation (e.g., K^+^) preserved the film’s intrinsic high electrical conductivity (1652 S cm^−1^), while the Seebeck coefficient was enhanced more than three-fold (16.5 μV K^−1^). As a result, a significantly improved room-temperature power factor of 44.98 μW m^−1^ K^−2^ was achieved. Importantly, the modified Ti_3_C_2_T_x_ films retained excellent flexibility, underscoring their potential for integration into flexible TE energy-harvesting devices. This study points to the vital necessity of surface termination control in bridging the gap between high electrical conductivity and improved Seebeck response in MXene-based TEs. More recently, Syamsai et al. (2024) [38] extended the family of TE MXenes by exploring tantalum carbide MXene (Ta_4_C_3_T_x_) for the first time (Figure 7e,f). Synthesized via hydrofluoric acid treatment to remove Al from the Ta_4_AlC_3_ MAX phase, the exfoliated Ta_4_C_3_T_x_ exhibited a Seebeck coefficient of 13.8 µV K^−1^ (Figure 7g) and a power factor of 1.88 µW m^−1^ K^−2^ at 803 K, along with a low lattice thermal conductivity of 5.42 W m^−1^ K^−1^. The material maintained stable TE performance over six thermal cycles, showing a high electrical conductivity of 400 S cm^−1^ (Figure 7h) and a weighted mobility of 1.02 cm^2^ V^−1^ s^−1^. These findings identify Ta_4_C_3_T_x_ as a thermally stable MXene with promising TE characteristics and provide valuable insights into engineering next-generation TE materials.

Collectively, these studies validate the scalability of MXene synthesis via etching and filtration methods and demonstrate the tunability of their TE properties through structural and surface engineering. However, challenges, such as the inherently high thermal conductivity and modest power factor in pure MXenes, continue to limit their overall TE efficiency. Consequently, increasing attention has turned to composite strategies where the high electrical conductivity of MXenes can be synergistically combined with secondary phases that suppress heat transport, offering a promising pathway toward high-performance TE materials.

### 3.2. MXene Composites for Enhanced TE Performance

The intrinsic limitations of pristine MXenes—particularly their relatively high thermal conductivity—have spurred significant efforts to develop MXene-based composites. By integrating MXenes with polymers, carbon-based materials, or inorganic TEs, researchers aim to achieve a favorable trade-off among high electrical conductivity, enhanced Seebeck coefficients, and suppressed thermal conductivity. Theoretical and experimental studies indicate that interfaces and heterostructures in such composites can serve dual roles: promoting energy carrier filtering and effectively scattering phonons. These mechanisms have enabled the development of MXene-containing TE materials with ZT values rivaling those of conventional systems such as Bi_2_Te_3_.

**Figure 8:**
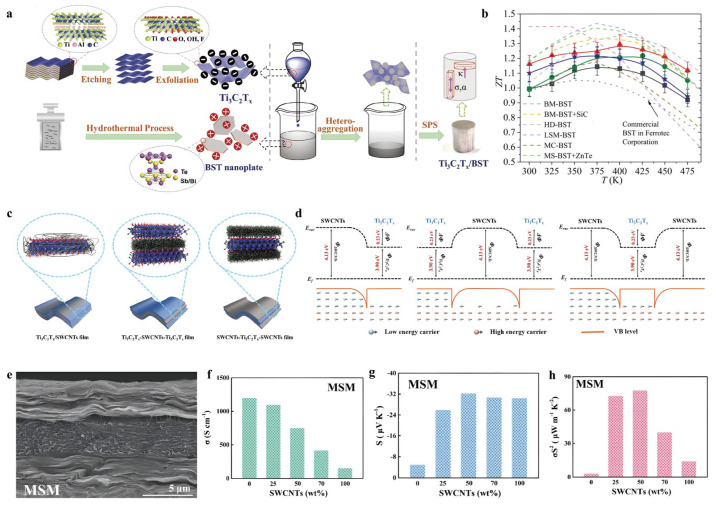
(**a**) Diagrammatic representation of the fabrication of Ti_3_C_2_T_x_ nanosheets and BST 2D platelets. Copyright 2019, WILEY-VCH Verlag GmbH [39]. (**b**) Temperature-sensitive ZT values of Ti_3_C_2_T_x_/BST composites in comparison with top-tier ZT value for p-type Bi_2_Te_3_-based alloys synthesized via various methods. Copyright 2019, WILEY-VCH Verlag GmbH [39]. (**c**) The schematic structure diagrams of Ti_3_C_2_T_x_/SWCNTs (M/S), Ti_3_C_2_T_x_–SWCNTs–Ti_3_C_2_T_x_ (MSM), and SWCNTs–Ti_3_C_2_T_x_–SWCNTs (SMS) films. Copyright 2021, Elsevier [40]. (**d**) Sowing energy-filtering effects. Copyright 2021 Elsevier [40]. (**e**) The schematic structure diagrams of Ti_3_C_2_T_x_–SWCNTs–Ti_3_C_2_T_x_ (MSM). Copyright 2021, Elsevier [40]. (**f**) Electrical conductivity of Ti_3_C_2_T_x_–SWCNTs–Ti_3_C_2_T_x_ (MSM). Copyright 2021 Elsevier [40]. (**g**) Seebeck coefficient Ti_3_C_2_T_x_–SWCNTs–Ti_3_C_2_T_x_ (MSM). Copyright 2021, Elsevier [40]. (**h**) Power factor of SWCNTs–Ti_3_C_2_T_x_–SWCNTs (SMS) films. Copyright 2021, Elsevier [40].

Recent advances have demonstrated that incorporating 2D MXene nanosheets into inorganic TE matrices is an effective approach to simultaneously optimize electrical and thermal transport behaviors. For instance, Lu et al. (2019) [39] introduced oxygen-terminated Ti_3_C_2_T_x_ MXene into a Bi_2_Te_3_-based (BST) matrix via a self-assembly strategy (Figure 8a). The resulting composite exhibited decoupled charge and phonon transport: electrical conductivity (690 S cm^−1^) was enhanced through hole injection, while the Seebeck coefficient (215 µV K^−1^) was maintained, resulting from interfacial potential barrier scattering. Furthermore, the aligned MXene layers served as efficient phonon barriers, markedly reducing the lattice thermal conductivity (0.32 W m^−1^ K^−1^). These synergistic effects led to a peak ZT of 1.3 and a record-high conversion efficiency of 7.8% under a 237 K temperature gradient, demonstrating the great promise of MXene/BST composites for TE power generation (Figure 8b).

Beyond inorganic TE composites, the rational construction of heterostructures combining MXenes with carbon-based materials has also demonstrated significant potential in optimizing TE properties. Ding et al. (2020) [40] fabricated a layered Ti_3_C_2_T_x_–SWCNTs–Ti_3_C_2_T_x_ composite film with a 2D–3D sandwich architecture using a wet-chemical assembly strategy (Figure 8c). This well-organized structure facilitated a high carrier concentration and the formation of double energy barriers (Figure 8d,e), enhancing carrier filtering while preserving high electrical conductivity (750.9 S cm^−1^) (Figure 8f) and an improved Seebeck coefficient (−32.2 µV K^−1^) (Figure 8g). The resulting power factor reached 77.9 µW m^−1^ K^−2^ at room temperature (Figure 8h), representing an approximately 25-fold enhancement over the pristine Ti_3_C_2_T_x_ films. This work exemplifies how precise heterointerface engineering can synergistically improve both charge transport and energy filtering, offering a promising design strategy for high-performance n-type TE materials based on MXenes and other 2D nanocomposites. In addition to structural engineering, interfacial energy modulation between p-type polymers and n-type MXenes has proven to be an effective approach for TE enhancement.

Extending this strategy, Jiang et al. (2021) [41] developed Ti_3_C_2_T_x_/SnTe nanocomposites through a solvothermal synthesis route. The addition of just 0.6 wt% MXene significantly suppressed intrinsic Sn vacancies, reduced the carrier concentration, and formed abundant heterointerfaces. These modifications improved both electrical and thermal transport properties, yielding a maximum ZT of approximately 0.63 at 823 K—an enhancement of 60% over pristine SnTe. This finding underscores the ability of even trace amounts of MXene to exert substantial influence on TE performance. In a similar vein, Zhao et al. (2024) [42] employed MXene as a nanoscale second-phase additive in Cu_2_Se-based systems. Using a hydrothermal method followed by vacuum hot-pressing, they demonstrated that only 0.2 mol% MXene was sufficient to induce a thinner, layered grain structure that promoted interlayer phonon scattering. This structural modulation reduced the thermal conductivity by up to 42%, reaching a minimum value of 0.54 W m^−1^ K^−1^. Simultaneously, interfacial band alignment between Cu_2_Se and MXene introduced an energy filtering effect, enhancing the Seebeck coefficient (230 µV K^−1^). These combined effects resulted in a high ZT of 1.77 at 923 K, marking a ~30% improvement compared to the undoped counterpart. Together, these studies underscore the versatility and efficacy of MXene as a multifunctional additive across various inorganic TE systems. Whether through phonon scattering, energy filtering, or defect regulation, MXene contributes to finely tuning TE properties via interface engineering. However, translating these promising results into real-world applications will require addressing remaining challenges, including material cost, long-term stability, and scalable fabrication techniques.

**Figure 9:**
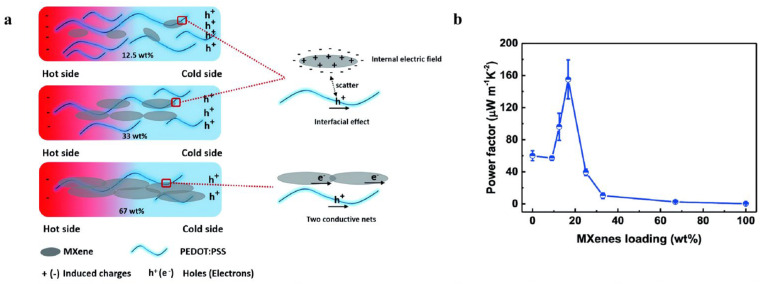
(**a**) Conceptual schematics of the interfacial interactions between MXene and PEDOT: PSS in composite systems with MXene content. Copyright 2020, American Chemical Society [43]. (**b**) TE properties of MXene/PEDOT: PSS composites depending on the MXene content, power factor. Copyright 2020, American Chemical Society [43].

Guan et al. (2020) [43] demonstrated that blending Ti_3_C_2_T_x_ MXene into the p-type polymer PEDOT: PSS resulted in a notable increase in the Seebeck coefficient from 23 to 57.3 μV K^−1^ and a corresponding rise in power factor to 155 μW m^−1^ K^−2^ (Figure 9b). This marks the first reported enhancement of a p-type polymer’s Seebeck coefficient via the incorporation of an n-type filler. This improvement is attributed to the formation of an internal electric field at the MXene–polymer interface caused by electron transfer from MXene to PEDOT: PSS, which acts as an energy filter by scattering low-energy carriers (Figure 9a). This strategy not only challenges the conventional design paradigm for polymer-based TEs, but also opens new possibilities for developing high-performance, flexible TE materials.

**Figure 10:**
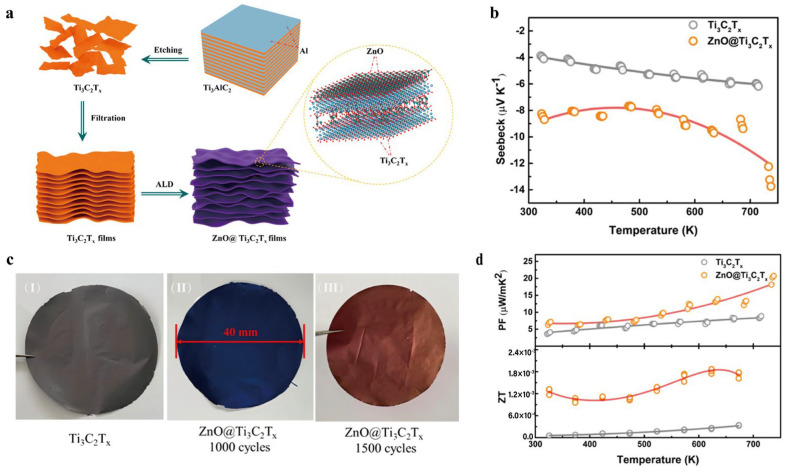
(**a**) Schematic illustration of the assembly of the ZnO@Ti_3_C_2_T_x_ composite films. Copyright 2022, American Chemical Society [44]. (**b**) Seebeck coefficient. Copyright 2022, American Chemical Society [44]. (**c**) The overall image of the ZnO@Ti_3_C_2_T_x_ film, and chromatic appearance progressively deepens with accumulating the ZnO cycle count. Copyright 2022, American Chemical Society [44]. (**d**) Power factor and ZT of the Ti_3_C_2_T_x_ and ZnO@Ti_3_C_2_T_x_ films. Copyright 2022, American Chemical Society [44].

Building upon these composite and interface engineering strategies, further advancements have been made through atomic-level structural design. Yan et al. (2022) [44] introduced atomic layer deposition (ALD) as a precise and controllable method to grow wide-bandgap ZnO layers onto Ti_3_C_2_T_x_ films, forming ZnO@Ti_3_C_2_T_x_ nanocomposites, and, with a rising number of ZnO deposition cycles, a gradual darkening of the film’s appearance is observed (Figure 10c). The conformal ZnO coatings formed a well-defined heterojunction with the underlying MXene, introducing a Schottky barrier that facilitated an effective energy-filtering effect (Figure 10a). This barrier selectively suppressed low-energy carriers, thereby significantly enhancing the Seebeck coefficient (−13.74 µV K^−1^) (Figure 10b). Concurrently, the sharp heterointerfaces induced strong phonon scattering, which reduced the thermal conductivity (4.99 W m^−1^ K^−1^) by nearly four-fold compared to pristine Ti_3_C_2_T_x_. As a result, the composite exhibited more than twice the power factor (21 μW m^−1^ K^−2^) and a considerable improvement in the overall ZT value (1.8 × 10^−3^) (Figure 10d). This ALD-enabled heterostructure design not only highlights the efficacy of atomic-level interface control in tuning electronic and thermal transport, but also provides a scalable and versatile strategy for fabricating next-generation 2D TE materials with finely tuned properties.

### 3.3. Flexible and Wearable TE Devices

The previous section explores various strategies for enhancing the TE properties of MXene-based composite materials, focusing on interface engineering and material hybridization. Building on this, the current section delves into the application of MXene materials in thin-film thermoelectric generators (TFTEGs), specifically in the form of p-n modules. These developments demonstrate the excellent TE performance and scalability of MXene thin films, establishing their potential for integration into wearable electronics and IoT sensors.

**Figure 11:**
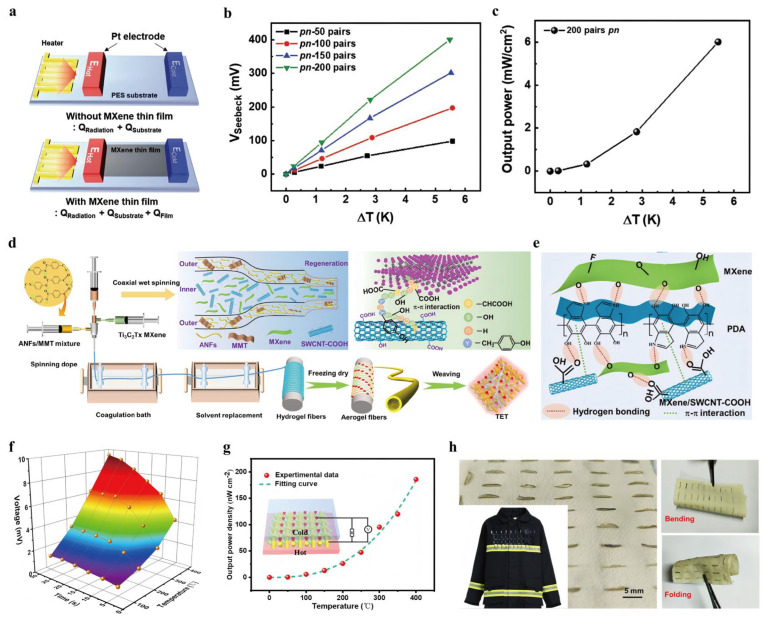
(**a**) Experimental setup of basal-plane thermal conductivity determination of MXene thin films. Copyright 2021, WILEY-VCH Verlag GmbH [45]. (**b**) Seebeck voltages of the MXene TFTEG in response to temperature lift. Copyright 2021, WILEY-VCH Verlag GmbH [45]. (**c**) Peak power output of the MXene-based TFTEG assembly featuring 200 pairs of p-n junctions relative to the temperature difference. Copyright 2021, WILEY-VCH Verlag GmbH [45]. (**d**,**e**) Coaxial wet spinning of p–n segment coaxial-structured TE fibers and the establishment of intermolecular forces (H-bonding and π–π stacking) among MXene, PDA and SWCNT-COOH. Copyright 2023, Springer Nature [47]. (**f**,**g**) Temperature-dependent voltage curves (100–400 °C) and the corresponding peak power density metrics of the as-synthesized TET system. Copyright 2023, Springer Nature [47]. (**h**) Visual representation of the flexible TET architecture (5 cm × 4.5 cm) constructed by embedding p–n-type segmented fibers within an aramid matrix. Copyright 2023, Springer Nature [47].

Park et al. (2021) [45] investigated the TE properties and scalability of MXene-based TFTEGs constructed using p-Mo_2_C and n-Mo_2_Ti_2_C_3_ thin films (Figure 11a). These films exhibited in-plane thermal conductivities of 0.37 W m^−1^ K^−1^ for p-Mo_2_C and 0.45 W m^−1^ K^−1^ for n-Mo_2_Ti_2_C_3_, with dimensionless figures of merit (ZT) of 1.7 × 10^−5^ and 2.6 × 10^−4^, respectively. Notably, the scalability of the MXene TFTEGs was demonstrated by the Seebeck voltage, which was Exhibited a linear dependence on the number of p-n modules. A TFTEG with 200 p-n modules generated a Seebeck voltage of 399.9 mV (Figure 11b) and an output power of 6 × 10^6^ nW cm^−2^ at a temperature difference of 5.4 K (Figure 11c), underscoring the promising potential of MXene-based TFTEGs for wearable electronic devices and IoT sensors. Similarly, Huang et al. (2022) [46] optimized the TE performance of Mo_2_TiC_2_T_x_ and Nb_2_CT_x_ MXenes through organic molecule intercalation and thermal treatment, controlling their behavior to yield n- and p-type materials. These optimized MXenes exhibited impressive TE power factors of 13.26 and 1.106 × 10^4^ µW m^−1^ K^−2^ at room temperature. An all-MXene flexible TENG was fabricated using Ti_3_C_2_T_x_ MXene as the electrical contact electrode due to its high conductivity (up to 8000 S cm^−1^). The nanogenerator, constructed with 20 p-n pairs, produced an output voltage of 35.3 mV and a power of 33.9 nW under a temperature difference of 30 °C. While the performance of the MXene-based TENG was lower than that of classical TE materials, it compared favorably with other solution-processed 2D material-based nanogenerators. Further optimization of the MXene’s composition and surface chemistry could improve efficiency for future applications in flexible energy harvesting devices.

He et al. (2023) [47] developed a self-powered fire warning system integrated into firefighting clothing, incorporating alternating p/n-type TE aerogel fibers made from n-type Ti_3_C_2_T_x_ MXene and p-type MXene/SWCNT-COOH, encapsulated in an aramid nanofiber protective shell (Figure 11d,e). These TE fibers enabled the creation of flexible, wearable fire warning devices that could sense temperatures ranging from 100 to 400 °C, generating a voltage of 7.56 mV and an output power density of 119.79 nW cm^−2^ at a 300 °C temperature difference (Figure 11f,g). The device was characterized by exceptional flame retardancy, breathability, and compatibility with body movement, positioning it as a promising solution for enhancing firefighter safety through its integration into firefighting clothing (Figure 11h).

**Figure 12:**
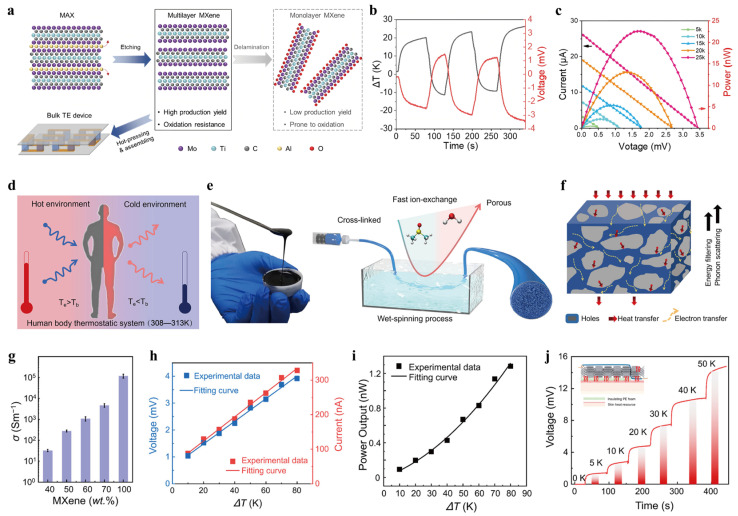
(**a**) Conceptual layout of the processing sequence for multilayer-MXene-based TE conversion devices. Copyright 2024, Royal Society of Chemistry [48]. (**b**,**c**) The temperature difference (DT) and open-circuit voltage (V_oc_) time-resolved of the device (at a constant cold-junction temperature of 293 K) and complemented by the electrical output and power-density curves evaluated under distinct temperature spans. Copyright 2024, Royal Society of Chemistry [48]. (**d**) Schematics depicting the temperature differential spanned by the human epidermis and the ambient atmosphere. Copyright 2023, American Chemical Society [49]. (**e**) Diagrammatic representation of the fabrication strategy for the 2D Ti_3_C_2_T_x_ based aerogel-like fibers using a coagulation-based spinning. Copyright 2023, American Chemical Society [49]. (**f**) Diagrammatic overview of the thermo-electronic transport channels embedded in the micro-architectured porous MP fiber. Copyright 2023, American Chemical Society [49]. (**g**) Electrical conductivity of the MP fibers with diverse MXene weight fractions within the 40–100% interval. Copyright 2023, American Chemical Society [49]. (**h**) Variation in the load voltage and output current in response to a range of temperature differences. Copyright 2023, American Chemical Society [49]. (**i**) Thermally driven peak power output of the TE module depending on the temperature gradient. Copyright 2023, American Chemical Society [49]. (**j**) Output voltage of the fiber-based TEG at different ΔT values. Copyright 2023, American Chemical Society [49].

Du et al.’s (2024) [48] research into the stability of non-exfoliated Mo_2_TiC_2_ and Mo_2_C stacks was scrutinized across various conditions, revealing that these multilayers surpass their monolayer analogs in both thermal robustness and anti-oxidation capacity (Figure 12a). Furthermore, evaluations of their thermoelectric behavior indicated that the as-synthesized multilayered forms maintain performance on par with delaminated sheets, with the economic viability, synthesis yield, and structural integrity being greatly improved. Moreover, to capture heat from physiological and ambient sources, a four-leg TE generator was constructed using MXene-based materials. This prototype, featuring two pairs of p/n-type multilayer MXene segments, delivered an open-circuit voltage of 3.43 mV across a temperature gradient of 25 K, with a peak power output of 22.68 nW (Figure 12b,c). Such findings highlight the potential of MXenes as economical and biocompatible candidates for the thermoelectric sector, particularly in the realm of self-powered wearable electronics.

To address the issue of high thermal conductivity, Li et al. (2023) [49] proposed a method to control the internal porosity of materials using a wet-spinning technique, which resulted in stretchable TE fibers (Figure 12d–f). This approach was applied to develop stretchable n-type TE fibers based on a hybrid of Ti_3_C_2_T_x_ MXene nanoflakes and polyurethane (MP). The fibers featured a 3D interconnected porous network that effectively reduced thermal conductivity while enhancing electrical conductivity and stretchability. The optimized MP-60 fibers, with 60 wt% MXene content, exhibited a high electrical conductivity of 12.5 S cm^−1^, a Seebeck coefficient of −8.3 μV K^−1^ (Figure 12g), and a low thermal conductivity of 0.19 W m^−1^ K^−1^, making them ideal for wearable bioelectronics applications such as low-grade body heat energy harvesting (Figure 12h–j). Zhang et al. (2023) [50] developed a novel MXene/CNT/PEDOT: PSS composite film for efficient respiration rate (RR) sensing, leveraging the TE effect. This composite material demonstrated significant advancements in mechanical strength, TE performance, and electromagnetic interference (EMI) shielding. The integration of carbon nanotubes (CNTs) enhanced the electrical conductivity (1002 S cm^−1^) between the MXene layers, resulting in a 5-fold increase in power factor (16.4 μW m^−1^ K^−2^) and a 2.4-fold improvement in tensile stress compared to pure MXene. Additionally, the film exhibited an impressive electromagnetic shielding efficiency of 59 dB, a 1.5-fold increase, and excellent self-heating capabilities. When applied to RR detection, this MXene-based composite showed significant potential for wearable health monitoring systems, offering multifunctional features such as respiration sensing, electromagnetic shielding, and self-heating, expanding MXene’s applications in the healthcare technology field.

These devices benefit from scalable fabrication methods like solution processing, but their low power density (<300 nW cm^−2^) limits applications to low-power wearables, and long-term stability under mechanical strain or humidity remains a concern. Nevertheless, the success of these flexible systems has inspired further exploration of multifunctional platforms, where MXenes’ TE capabilities are combined with sensing or energy storage to create integrated solutions for wearable electronics and health monitoring applications.

### 3.4. Multifunctional MXene-Based TE Systems

The versatility of MXenes, demonstrated in flexible TE devices, has spurred the development of multifunctional systems that integrate energy harvesting with sensing, energy storage, actuation, or thermal management. These integrated platforms aim to achieve compact, efficient, and self-powered solutions for next-generation wearable electronics, soft robotics, and smart textiles. Leveraging the unique electrical conductivity, thermal response, and surface functionalization potential of MXenes, researchers have begun to design thermally chargeable supercapacitors (TCSCs), light-responsive actuators, and multimodal sensors that not only harvest low-grade heat, but also perform other essential functions in situ. The following representative studies exemplify this multifunctional integration trend and highlight the ongoing innovation in MXene-based flexible TE systems.

**Figure 13:**
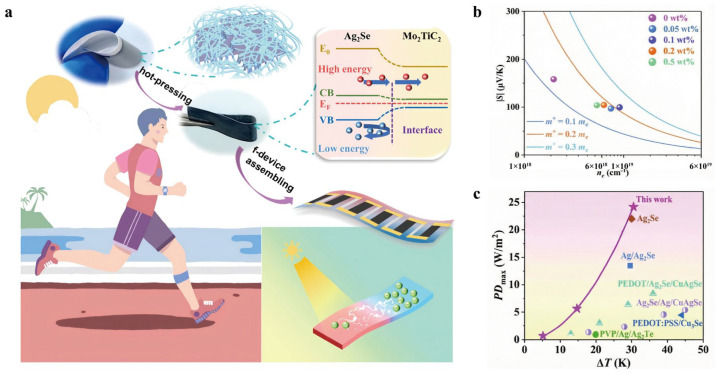
(**a**) Diagrammatic protocol and underlying microscopic interplay during the construction of Ag_2_Se/MXene composite architectures and representative TE device units. Copyright 2024, Royal Society of Chemistry [51]. (**b**) Experimentally determined Seebeck coefficients relative to SPB-modeled curves. The fitting line is based on a single parabolic band approximation, incorporating the DOS effective mass (m*). Copyright 2024, Royal Society of Chemistry [51]. (**c**) Comparative performance landscape: PDmax of the current work versus other f-TEG counterparts. Copyright 2024, Royal Society of Chemistry [51].

Qin et al. (2024) [51] introduced a nano compositing strategy involving 2D MXene nanosheets as fillers into a silver selenide (Ag_2_Se) nanowire matrix (Figure 13a). The synergy between the highly crystalline Ag_2_Se grains and the distinctive layered architecture of the MXene significantly boosted the power factor and mechanical toughness of the resulting films (Figure 13b). Internal mechanism investigations revealed that the MXene sheets act as conductive bridges. This bridge not only speeds up the charge transfer between nanowires, but also makes the whole structure more robust. Experiments showed the film hitting a room temperature power factor of 2.125 × 10^9^ nW m^−1^ K^−2^, peaking at 3.109 × 10^9^ n W m^−1^ K^−2^ at 400 K. Even after 3000 bends at a 4 mm radius, it kept 93% of its conductivity. An f-TEG comprising six legs was fabricated using this optimized film. The device delivered a maximum power density of 2.42 × 10^6^ nW cm^−2^ under a temperature gradient of 31 K, confirming its robust energy-harvesting capacity (Figure 13c).

**Figure 14:**
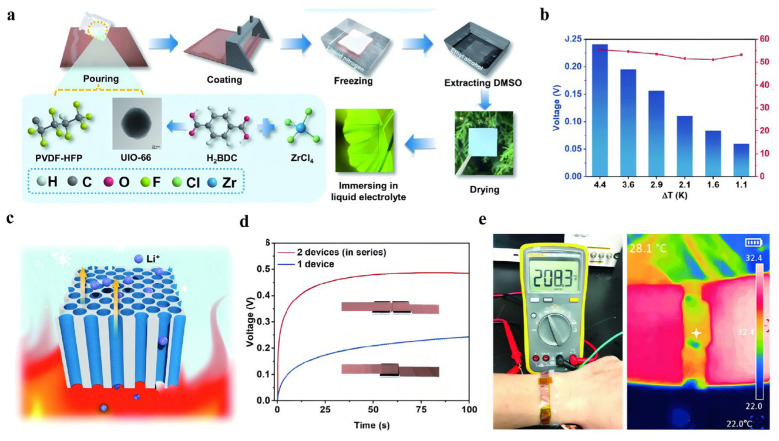
(**a**) Flow diagram of the synthetic process and gelation of PVDF-HFP@UIO-66 (PHU) membrane. Copyright 2024, WILEY-VCH Verlag GmbH [52]. (**b**) Thermal voltage and corresponding Seebeck coefficients of TCSC at different temperature differences. Copyright 2024, WILEY-VCH Verlag GmbH [52]. (**c**) Schematic of thermal diffusion effect of Soret mechanism. Copyright 2024, WILEY-VCH Verlag GmbH [52]. (**d**) Thermal voltage comparison between a single TCSC and two TCSCs in series [52]. (**e**) Simulate thermal charging in real-life scenarios. Copyright 2024, WILEY-VCH Verlag GmbH [52].

Similarly, Chen et al. (2024) [52] reported a high-performance and flexible TCSC using ZMO@Ti_3_C_2_T_x_ MXene composite electrodes and a UIO-66-doped multichannel PVDF-HFP ionogel electrolyte (Figure 14a). The device displayed a Seebeck coefficient of 5.54 × 10^4^ µV K^−1^ and a heat-to-electricity conversion efficiency of 6.48% at a ΔT of just 4.4 K, while maintaining excellent cycling stability under both high (3 K) and low (1 K) temperature differences (Figure 14b). The enhanced performance was attributed to the synergistic effects of a freeze-cast multichannel ionogel architecture for accelerated ion migration, high ion adsorption by UIO-66 MOF, and proton-rich ZMO@Ti_3_C_2_T_x_ structures that facilitated efficient charge carrier transport (Figure 14c). When applied to a wearable scenario, a single TCSC unit worn on the arm generated 208.3 mV (Figure 14e), and two units connected in series achieved 500 mV (Figure 14d), demonstrating the device’s strong potential for harvesting low-grade heat from the human body to power wearable systems.

**Figure 15:**
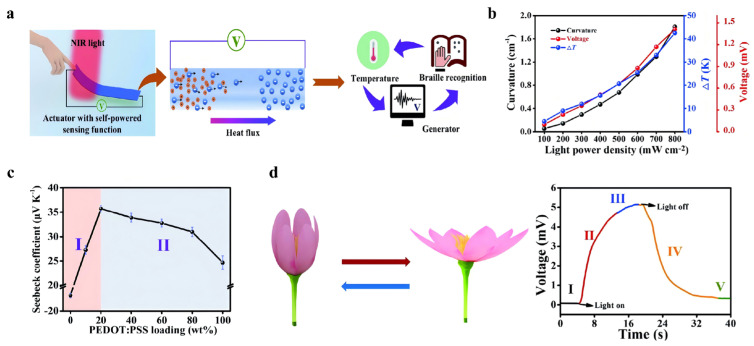
(**a**) Diagrammatic overview of the working principles and practical application of the actuator with intrinsic self-powered sensing capabilities. Copyright 2023, Royal Society of Chemistry [53]. (**b**) The irradiance-dependent mechanical bending response, photothermal gradient, and peak thermoelectric potential of the PEDOT: PSS/MXene/PI transducer. Copyright 2023, Royal Society of Chemistry [53]. (**c**) Seebeck coefficient of then PEDOT: PSS/MXene film depending on the loading weight content of the PEDOT: PSS. Copyright 2023, Royal Society of Chemistry [53]. (**d**) Diagrammatic representation of the opening and closing states of a bionic flower. Copyright 2023, Royal Society of Chemistry [53].

Expanding from TE storage to multifunctional actuation and sensing, Qian et al. (2023) [53] fabricated a light-driven flexible actuator with integrated self-powered sensing capabilities by coupling a PEDOT: PSS/MXene composite layer with a polyimide (PI) substrate (Figure 15a). The device achieved a high bending curvature of 1.8 cm^−1^ under near-infrared light (8 × 10^8^ µW cm^−2^ for 10 s), driven by the mismatch in photothermal expansion between the two layers (Figure 15b). The actuator also exhibited a Seebeck coefficient of 3.57 × 10^4^ µV K^−1^, which was attributed to interfacial energy filtering and synergistic carrier transport between PEDOT: PSS and MXene (Figure 15c). Notably, the output voltage dynamically correlated with mechanical deformation, enabling real-time, self-powered motion monitoring. Demonstrative applications included a bionic flower capable of light-triggered blooming and status feedback (Figure 15d), as well as a smart Braille display system that combined tactile recognition with electrical signal output, highlighting the actuator’s potential in assistive technologies, self-powered soft robotics, and intelligent human–machine interfaces.

**Figure 16:**
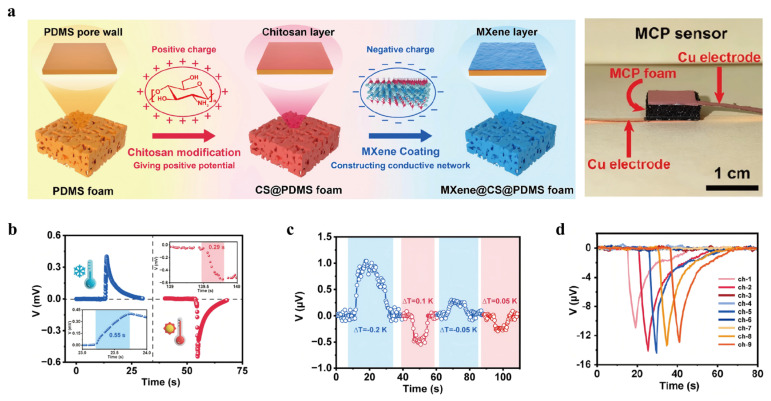
(**a**) Schematic illustration of the fabrication of MCP foam. Copyright 2023, American Chemical Society [54]. (**b**) Dynamic response profiles of the MCP sensor toward bipolar thermal stimuli (high vs. low temperatures). Copyright 2023, American Chemical Society [54]. (**c**) Voltage response of the MCP sensor at an incremental thermal bias of 0.05K. Copyright 2023, American Chemical Society [54]. (**d**) Multichannel voltage outputs/resistance changes in the multimode input terminal to identify the numerical input of “12589” by noncontact sensing. Copyright 2023, American Chemical Society [54].

In another direction, Gao et al. (2023) [54] designed a dual-mode MXene-based sensor for multimodal sensing capable of independently detecting temperature and pressure without signal interference (Figure 16a). This was achieved by integrating Ti_3_C_2_T_x_ MXene sheets onto porous and elastic substrates, leveraging both the TE (Seebeck) effect for temperature sensing and the piezoresistive effect for pressure detection (Figure 16b). The sensor demonstrated a minimum thermal resolution of 0.05 K, superior signal clarity, rapid feedback time, and excellent mechanical durability (Figure 16c). Its practical use was validated by its integration into flexible sensing platforms and a multifunctional interface (Figure 16d), where machine learning-based data processing enabled 100% classification accuracy, underscoring its promise for next-generation robotic sensing and human–machine interface applications.

Table 1 systematically summarizes the main research progress of MXene-based thermoelectric materials in recent years, covering pure phase MXene, composites of MXene and traditional thermoelectric materials, and hybrid systems of MXene and polymer/carbon nanotubes. The data show that pure MXene (Mo_2_TiC_2_T_x_, Ti_3_C_2_T_x_) typically exhibits extremely high conductivity (up to the order of 10^3^–10^6^ S cm^−1^), but its Seebeck coefficient is generally low (<50 μV K^−1^), leading to a limited power factor. By compounding with high-efficiency bulk thermoelectric materials (such as SnTe, Cu_2_Se, Ag_2_Se, etc.), the Seebeck coefficient and overall TE efficiency of the system can be markedly enhanced. For example, the Ti_3_C_2_Tx/SnTe composite achieves a high-power factor of ~2000 μW m^−1^ K^−2^ at 823 K, while the Cu_2_Se/MXene system achieves a ZT value of 1.77 at 923 K, demonstrating potential in high-temperature thermoelectric applications.

Attention should be drawn to the fact that flexible thermoelectric materials have become an important development direction. By combining MXene with flexible conductive polymers (PEDOT: PSS), carbon nanotubes (SWCNTs), or constructing multilayer heterostructures, it is possible to obtain acceptable thermoelectric outputs (PF = 155 μW m^−1^ K^−2^ for MXene/PEDOT: PSS, ZT = 0.12) while maintaining good mechanical flexibility. In addition, some studies have effectively improved the Seebeck coefficient through elemental doping or surface modifications, such as modification of Ti_3_C_2_T_x_, further optimizing the power factor.

However, most current studies still focus on improving electrical transport performance, and the thermal conductivity data is relatively missing; the in-plane/out-of-plane thermal conductivity characterization for devices especially needs to be strengthened. Future research needs to systematically characterize thermal conductivity and calculate ZT values while focusing on the performance stability of materials in a wide temperature range (from room temperature to above 900 K), interface engineering, and large-scale preparation processes to facilitate the technological utilization of MXene-based TE materials.

### 3.5. Correlation and Discrepancy Analysis: From Theory to Experiment

To address the consistency between theoretical predictions and practical outcomes, we present a systematic comparison of representative MXene systems in Table 2. While Section 2 highlights the idealized potential of these 2D materials, the experimental values discussed in Section 3 reflect the complexities of material synthesis and device integration.

The systematic comparison reveals that experimental values often differ from theoretical ideals due to several physical factors identified in recent research:

1. Surface Chemistry Complexity: Theoretical models typically assume uniform surface terminations like pure -O. However, experimental MXenes feature a stochastic mixture of -F, -OH, and -O groups, which alter the Fermi level and reduce the Seebeck response.
2. Dynamic Structural Evolution: Experimental studies observed that, above 500 K, the deintercalation of water and organic molecules significantly improves interlayer coupling, leading to a surge in electrical conductivity not captured in static DFT models.
3. Microstructural Defects: Actual MXene samples contain lattice disorders, dislocations, and nanosized pores. These features introduce additional Umklapp and defect scattering, which effectively lowers the thermal conductivity (κ) compared to perfect crystal predictions.

## 4. Conclusions and Challenges

In this review, we comprehensively summarize the experimental progress and theoretical insights related to the use of MXene-based materials for flexible TE energy harvesting. MXenes, as a novel class of 2D transition metal carbides, nitrides, and carbonitrides, have shown remarkable promise due to their high electrical conductivity, tunable surface terminations, and intrinsic mechanical flexibility. Their ability to form composites and heterostructures with diverse materials further enhances their suitability for integration into next-generation flexible TEGs.

Compared to other established flexible TE materials, MXenes occupy a unique niche. Unlike conducting polymers (e.g., PEDOT: PSS), which excel in low thermal conductivity but often suffer from inferior electrical conductivity and long-term stability, MXenes provide metallic-like conductivity and superior power factors. When compared to carbon-based materials (such as CNTs and graphene), MXenes offer lower intrinsic thermal conductivity and more diverse chemistry for work-function tuning via surface terminations (OH, –O, –F), although carbon materials typically exhibit higher environmental stability. Furthermore, while transition metal dichalcogenides (TMDs) and other 2D semiconductors often possess higher Seebeck coefficients, MXenes outperform them in terms of hydrophilicity and solution-processability, enabling easier integration into large-area flexible substrates. However, the primary disadvantage of MXenes remains their relatively low Seebeck coefficient and vulnerability to oxidation, which currently lags the robust performance of some inorganic polymer hybrids.

We examined a wide range of experimental strategies—including surface engineering, compositional tuning, and hybridization with polymers, carbon-based materials, and inorganic semiconductors—that have significantly advanced the TE performance of MXene-based systems. These efforts have successfully demonstrated enhanced power factors, reduced thermal conductivity, and improved mechanical compatibility. Moreover, the development of flexible and wearable MXene-based TE devices has opened new avenues for self-powered electronics, health monitoring, and human–machine interaction technologies.

Despite these advances, several fundamental and practical challenges persist. First, the relatively low Seebeck coefficients and high thermal conductivities of pristine MXenes limit their standalone TE efficiency. Second, environmental instability, particularly oxidation in ambient conditions, remains a significant barrier to long-term device reliability. Third, the scalable and uniform synthesis of high-quality MXenes with controlled surface chemistry is still a bottleneck for industrial applications. Furthermore, a deeper understanding of the interplay between surface functional groups, interlayer interactions, and carrier transport mechanisms is required to unlock the full potential of MXene-based TE materials. Beyond technical performance, economic and industrial viability remains a critical hurdle. The high cost of precursors (MAX phases) and the reliance on hazardous, expensive etching agents like hydrofluoric acid (HF) pose significant challenges for large-scale adoption. Furthermore, reproducibility is often compromised by batch-to-batch variations in flake size, surface functionalization, and defect density, which are sensitive to subtle changes in etching time and temperature. From a manufacturing perspective, integrating MXenes into existing industrial lines—such as CMOS processes or standard textile finishing—requires overcoming compatibility issues related to solvent toxicity and the high-temperature stability of the organic components in the composites.

Looking forward, we propose several promising directions for future research:

1. Advanced Surface and Interface Engineering: Precise modulation of surface terminations and interlayer spacing via chemical or physical treatments could be leveraged to engineer electronic band structures and phonon scattering processes. Exploring new functional groups and interface chemistry may lead to significant improvements in both Seebeck coefficient and thermal conductivity suppression.
2. Heterostructure and Composite Design: Constructing MXene-based heterostructures with other 2D materials or organic frameworks can enable energy filtering, phonon blocking, and mechanical reinforcement. In particular, the development of Janus MXenes, double transition metal systems, and strain-engineered architecture represents an exciting frontier for high-ZT materials.
3. Integration with Flexible Architectures: Embedding MXenes into fibers, textiles, or stretchable films will be essential for realizing wearable TE devices. Strategies such as wet spinning, vacuum filtration, and 3D printing should be explored for scalable manufacturing. Additionally, the coupling of TE functionality with other modalities—such as sensing, actuation, and energy storage—can yield multifunctional platforms for smart wearables and soft robotics.
4. Machine Learning and High-Throughput Screening: The application of data-driven approaches to screen and predict MXene candidates with optimal TE properties can accelerate material discovery. Coupling machine learning with experimental validation may significantly shorten the development cycle for new materials.
5. Device-Level Optimization: Future efforts should also focus on improving the architecture and thermal management of MXene-based TEGs. This includes enhancing contact interfaces, optimizing module layout, and improving thermal gradients to increase overall power output. For real-world deployment, stability under bending, humidity, and elevated temperatures must be systematically addressed.
6. Sustainable and Scalable Manufacturing: Future research must prioritize the development of green, HF-free synthesis methods to reduce environmental impact and cost. To ensure reproducibility, establishing standardized protocols for material characterization and processing is essential. Moreover, exploring roll-to-roll processing, screen printing, and other high-throughput manufacturing techniques will be vital for integrating MXene-based TEGs into the existing electronics and garment industries at a commercial scale.

In conclusion, MXenes hold tremendous potential as next-generation materials for flexible TE applications. Their unique combination of electrical conductivity, structural tunability, and solution-processability offers a rich platform for both fundamental research and practical device engineering. Continued interdisciplinary efforts that bridge materials science, device physics, and scalable fabrication will be vital to transforming MXene-based TE systems from laboratory prototypes to real-world technologies.

## Figures and Tables

**Table 1:** Summary table of performance parameters of different thermoelectric materials and their composites.

	Seebeck (μV K^−1^)	σ (S cm^−1^)	Power Factor PF (μW m^−1^ K^−2^)	κ (W m^−1^K^−1^)	ZT	Flexibility	Test Temperature (K)	Reference
Mo_2_TiC_2_T_x_	−47.3	1380	309	—	—	no	803	Kim et al. (2017) [37].
Ti_3_C_2_T_x_ (Pristine)	~−10	1652	<15	—	—	no	300	Liu et al. (2020) [13].
Ti_3_C_2_T_x_ (Modified)	~−35	1652	44.98	—	—	yes	300	Liu et al. (2020) [13].
Ta_4_C_3_T_x_	13.8	4000	1.88	5.42	—	no	803	Syamsai et al. (2024) [38].
MXene/BST	~215	690	~25	0.32	1.3	no	400	Lu et al. (2019) [39].
Ti_3_C_2_T_x_/SnTe	156	6000	~2000	~8	0.63	no	823	Jiang et al. (2021) [41].
Cu_2_Se/MXene	~230	—	~1300	0.54	1.77	no	923	Zhao et al. (2024) [42].
Ti_3_C_2_T_x_-SWCNTs-Ti_3_C_2_T_x_	−32.2	750.9	77.9	—	—	yes	298	Ding et al. (2020) [40].
MXene/PEDOT: PSS	57.3	736.4	155	0.35	0.12	yes	298	Guan et al. (2020) [43].
ZnO@ Ti_3_C_2_T_x_	−13.74	1610	~21	4.99	~1.8 × 10^−3^	yes	625	Yan et al. (2022) [44].
p-Mo_2_C	308	22	—	0.37	1.7 × 10^−5^	yes	RT △T = 5.4k	Park et al. (2021) [45].
n-Mo_2_Ti_2_C_3_	−25	62,800	—	0.45	2.6 × 10^−4^	yes	RT △T = 5.4k	Park et al. (2021) [45].
p-Nb_2_CT_x_	−2	805.1	11.06	—	—	yes	633.15	Huang et al.(2022) [46].
n-Mo_2_TiC_2_T_x_	−30	200	13.26	—	—	yes	633.15	Huang et al.(2022) [46].
p-MXene/SWCNT-COOH	33,000	120	0.179	—	—	yes	623.15	He et al. (2023) [47].
n-Ti_3_C_2_T_x_ MXene	−11,000	830	0.098	—	—	yes	623.15	He et al. (2023) [47].
Mo_2_C	45,000	0.25	0.1	0.206	1.3	yes	300	Du et al. (2024) [48].
Mo_2_TiC_2_	−22,500	23.43	1.1867	1.355	—	yes	300	Du et al. (2024) [48].
Ti_3_C_2_T_x_ MXene	8.3	1.25 × 10^6^	0.086	0.19	—	yes	300	Li et al. (2023) [49].
MXene/CNT/PEDOT: PSS	9	1938	16.4	—	—	yes	300	Zhang et al. (2023) [50].
Ag_2_Se/MXene	−109	1800	2125	0.92	0.59–1.33	yes	300	Qin et al. (2024) [51].
ZnMn_2_O_4_@Ti_3_C_2_T_x_ MXene	55.4 × 10^3^	2.82	—	—	—	yes	—	Chen et al. (2024) [52].
PEDOT: PSS/MXene (Ti_3_C_2_T_x_)	35.7 × 10^3^	—	—	—	—	yes	300	Qian et al. (2023) [53].
Ti_3_C_2_T_x_ MXene	−32.2	750.9	77.9	—	—	yes	298	Gao et al. (2023) [54].

**Table 2:** Systematic comparison of theoretical predictions vs. experimental validations for MXene TE materials.

Material System	Property	Theoretical Prediction (DFT/Boltzmann)	Experimental Result	Primary Reference Linkage
Mo-based MXenes	PF	Predicted 1.3 × 10^16^ µW m^−1^ K^−1^ at 600 K for Mo_2_C monolayers	~1 × 10^2^ µW m^−1^ K^−2^ at 803 K for Mo_2_CT_x_ films	Khazaei (2014) [19] vs. Kim (2017) [37]
Ti-based MXenes	S	Oxygen termination on Ti_2_C increases S up to ~350 µV K^−1^	Alkali-treated Ti_3_C_2_T_x_ (-O term) enhanced S to 16.5 µV K^−1^	Guo (2018) [23] vs. Liu (2020) [13]
Nb-based MXenes	κ	Strong electron-phonon (e-p) scattering induces reduction in κ	PF of 11.06 µW m^−1^ K^−2^ achieved for p-type Nb_2_CT_x_	Huang (2019) [22] vs. Huang (2022) [46]

## Data Availability

No new data were created or analyzed in this study. Data sharing is not applicable to this article.
